# Socially-mediated arousal and contagion within domestic chick broods

**DOI:** 10.1038/s41598-018-28923-8

**Published:** 2018-07-12

**Authors:** Joanne L. Edgar, Christine J. Nicol

**Affiliations:** 1 Bristol Veterinary School, University of Bristol, Langford House, Langford BS40 5DU UK; 2 Royal Veterinary College, Hawkshead Lane, North Mimms, Herts AL9 7TA UK

## Abstract

Emotional contagion – an underpinning valenced feature of empathy – is made up of simpler, potentially dissociable social processes which can include socially-mediated arousal and behavioural/physiological contagion. Previous studies of emotional contagion have often conflated these processes rather than examining their independent contribution to empathic response. We measured socially-mediated arousal and contagion in 9-week old domestic chicks (n = 19 broods), who were unrelated but raised together from hatching. Pairs of observer chicks were exposed to two conditions in a counterbalanced order: air puff to conspecifics (AP) (during which an air puff was applied to three conspecifics at 30 s intervals) and control with noise of air puff (C) (during which the air puff was directed away from the apparatus at 30 s intervals). Behaviour and surface eye temperature of subjects and observers were measured throughout a 10-min pre-treatment and 10-min treatment period. Subjects and observers responded to AP with increased freezing, and reduced preening and ground pecking. Subjects and observers also showed reduced surface eye temperature - indicative of stress-induced hyperthermia. Subject-Observer behaviour was highly correlated within broods during both C and AP conditions, but with higher overall synchrony during AP. We demonstrate the co-occurrence of socially-mediated behavioural and physiological arousal and contagion; component features of emotional contagion.

## Introduction

Emotional contagion^1^ has been portrayed as a simple or automatic affective process^2–5^ that may (together with other more complex processes) underpin a full capacity for empathy^6^. Decety and Jackson^7^ define emotional contagion as sharing emotion without self-awareness. Until recently, studies of emotional contagion focussed only on humans but in recent years there has been a growing interest in assessing the foundational underpinnings of empathy in animals. Many studies now purport to investigate emotional contagion (pigs^2,8^; bonobos^3^; mice^4^; dogs^9,10^). However, an emotional state is, by definition, one that is either positive or negative; the individual will either work to achieve such a state or will work to avoid or escape it^11–14^. Therefore, to measure the transfer of emotion, experiments must conclusively demonstrate a valenced response in both subjects and observers (see^15^ for a review). Such independent evidence is rarely obtained (although see^16^), making the study of emotional contagion very difficult. We suggest that the difficulty of measuring emotional contagion is also compounded by the frequent conflation of the simpler social processes that contribute to emotional contagion, meaning that researchers interested in emotional contagion may be studying diverse phenomena. In the current study we focus on this issue, showing two components of emotional contagion that are conceptually distinct and empirically separable.

One process is that of socially-mediated arousal (SMA), the increased sensory alertness, attention and readiness to respond that may occur when one animal witnesses a conspecific’s behaviour or physiology. The response that is produced does not necessarily a) have to match (e.g. an observer could show freezing in response to a subject’s flight behaviour or vice versa) or b) be associated with either positive or negative valence. Behavioural indicators of SMA include specific freezing responses (a parasympathetic brake on the nervous system, relevant to enhanced perception and action preparation^17^) when observing conspecifics distressed by electric shock^4,18^ or restraint^8^ and behavioural shifts away from maintenance behaviours such as preening and towards vigilance when observing offspring mildly distressed by air puffs^19^. Physiological components of socially-mediated arousal are less studied, but include increased heart rate during aggressive encounters (geese^20^) along with stress-induced hyperthermia in birds when watching mildly distressed offspring (chickens^19,21^). Although these arousal processes are not necessarily accompanied by a valenced emotional response, they can incorporate relatively complex cognitive appraisals, such that observers react with increased arousal to the conspecific’s situation even in the absence of conspecific distress^22^.

Another process that can occur as part of the broader process of emotional contagion is behavioural/physiological contagion/mimicry, which occurs when one animal matches the behavioural or physiological response of another. As with arousal, behavioural contagion is not necessarily associated with a valenced emotional component and can be measured using markers such as increased behavioural synchrony in a range of situations, including those that are seemingly positive (play in keas^23^; rapid mimicry of facial expression in response to play in dogs^24^), neutral (contagious yawning in monkeys^25^ and chimps^26^); and negative (contagion of agonistic vocalisations in marmosets^27^; socially-induced flight reactions in pigeons^28^; vigilance in sheep^29^) for the conspecific. Physiological contagion is less studied, but has been shown in the context of cortisol covariation during stressful situations in humans^30,31^, songbirds^32^ and teleost fish^33^.

The extent to which SMA and behavioural/physiological contagion are associated has, to our knowledge, received no empirical attention. Whilst behavioural and/or physiological contagion may follow directly from SMA, the two processes may not necessarily always follow this causal relationship. In some circumstances behavioural/physiological contagion could precipitate rather than follow SMA. For example, an individual pigeon may innately follow members of the flock in taking flight, and only subsequently show signs of conditioned arousal caused by the sight and/or context of fleeing conspecifics. In other situations, increased arousal may not be accompanied by behavioural or physiological contagion at all. Our previous studies using a hen-chick model have pointed to the occurrence of SMA in the absence of behavioural synchrony^19,22^. Indeed, signs of offspring distress may cause a parent bird to increase attentiveness towards the brood leading to a protective aggressive response to predators or conspecifics, whilst chimpanzees^34^ and horses^35^ may mount a consolation response to a defeated conspecific. Similarly, behavioural contagion can occur in the absence of SMA; contagious yawning in dogs, for example, is not associated with altered heart rate^36^.

Given the complex relationship between the two social processes, it is surprising that it is only occasionally acknowledged that arousal and behavioural/physiological contagion are distinct processes, which can occur separately or together^15,24,37^. If we are to study emotional contagion as an underpinning attribute of empathy then it is essential that we characterise its component parts and processes, defined at species and context-specific level. Previously, we demonstrated the existence of SMA within the mother-offspring bond in chickens. Whilst watching their chicks being exposed to aversive air puffs, mother hens responded with increased heart rate, stress-induced hyperthermia, increased vocalisations, standing alert and reduced preening^19^. There were no differences in the responses of hens who had or had not already received prior (individual, but in the presence of their chicks) experience of air puffs. Using the same methodology, we found no evidence that adult hens showed SMA when witnessing familiar, but non-related adults receiving air puffs, regardless of whether they had individual prior experience of the air puffs^21^).

Chickens have proved a useful model in understanding some of the foundational processes underpinning empathy, but further work is needed to understand why such variable responses are observed in birds that differ in age, reproductive status, familiarity and experience. In the present study we aimed to determine the extent to which domestic chicks, raised together from hatching, showed SMA, demonstrated by behavioural and physiological changes indicative of increased alertness and attentiveness and behavioural and physiological contagion measured by group behavioural and physiological synchrony. Clarification of the relationship between these two processes will facilitate future work on emotional contagion.

## Materials and Methods

### Ethics statement

This project was carried out following ethical approval by the University of Bristol (University Investigation Number: UB/14/054) and in accordance with Association for Study of Animal Behaviour (ASAB) ethical guidelines. At the end of the study all animals were rehomed to responsible smallholders.

### Animals and housing

19 broods of five female chicks (unrelated Lohmann classic) were each hatched under a broody hen housed in a floor pen (1.5 m × 1 m) (see Supplementary Information for full details).

### Habituation to test box

In their groups of 5, the chicks were gradually habituated to handling and being placed into a test box. The test apparatus was a 100 cm × 50 cm wooden structure divided into two sections; the Subject box and the Observer box, which were separated by a wire mesh screen. Wire panels on the front of the boxes facilitated thermal video recording. (See Supplementary Information for figure and habituation schedule). Following habituation chicks were used in a separate study to determine the influence of natural variation in maternal care on chick development (see Supplementary Information).

### Testing

Testing began when the chicks were nine weeks old. Within each pen of five chicks, two chicks were randomly assigned the role of observers and three chicks were assigned the role of subjects. Of the 19 chick broods, 10 were pre-assigned to receive AP on the first testing day and C on the second, and 9 were pre-assigned to receive C on the first testing day and AP on the second.

### Settling period

On the day of testing, the 2 observer chicks were first collected from their home pen and placed into the ‘Observer’ box. The 3 subject chicks were immediately collected from the home pen and placed into the ‘Subject’ box. Following a 5-minute settling period, a 20-minute test period began.

### Test period

The test period began with a 10-minute pre-treatment period in which no air puff was sprayed, immediately followed by a 10-minute treatment period in which either of the following two treatments were applied:

Control with Noise (C) – An air puff was sprayed from the same location as AP (see below) but was directed away from both the Subject and Observer boxes, so that the chicks were exposed to the noise of the air puff (identical to the sound of a household aerosol being sprayed). This occurred for one second every 30 seconds.

**Figure 1:**
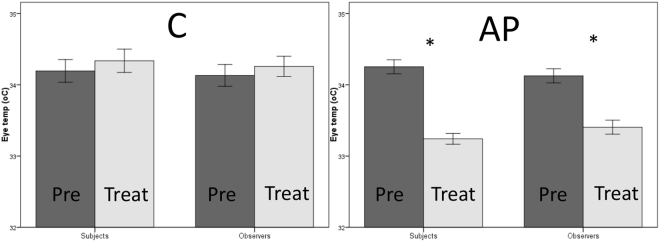
Eye temperature response of Subjects and Observers to Control (C) and Air puff (AP) during the pre-treatment (Pre) and treatment (Treat) periods.

Air puff to subject (AP) - An air puff from a canister of inert compressed air (Sprayduster, AF International, UK) was sprayed into the Subject box for one second every 30 seconds (see Fig. 1 Supp for location and direction of air puff).

### Physiological and behavioural measures

Throughout the pre-treatment and treatment periods behaviour and eye temperature were recorded using a thermal video camera (FLIR SC305) positioned one metre from the test box. The thermal camera was set to an emissivity of 0.96 and the ambient temperature of the testing room was maintained at 20 °C.

Behaviours and eye temperatures were later extracted using FLIR ResearchIR Software version 1.2. Behaviour was extracted using one-minute scan sampling; recording the behaviour of each of the five chicks every one minute (see Table Supp2 for ethogram), 10 seconds after the air puff during AP, or matched control point for C. Eye temperature was recorded at the same timepoint as behaviour. For extraction of eye temperature, a time window of ten seconds at either side of the one-minute mark was allowed to ensure that a clear image of the side of each chick’s head was obtained.

### Statistical analyses

Eye temperature data were first averaged to give one data point for the subjects and one data point for the observers for each minute during the pre-treatment and treatment periods. Then we calculated the mean temperature for the whole 10 min pre-treatment and 10 min treatment periods for both subjects and observers, giving one data point for each treatment (C and AP) for subjects and one data point for observers. To determine SMA, a mixed between-within subjects repeated measures ANOVA was conducted with period (Pre-treatment and treatment) and treatment (C and AP) as within-subjects’ factors and the order of testing (C before AP and AP before C) and chick identity (Subject or Observer) as between-subjects’ factors. To assess synchrony, pearson correlations were used to determine whether within-brood subjects’ and observers’ eye temperature responses during the AP and C were correlated.

For behaviour, the number of chicks performing each behaviour (see Supplementary Information for ethogram) was recorded every minute. Then, the percentage of observations spent performing each behaviour was calculated for the whole 10 min pre-treatment and 10 min treatment periods for both subjects and observers, giving one data point for each treatment for subjects and one data point for observers. Behaviour data were non-normally distributed (and were not improved by log or square root transformation) and so, to determine SMA, behaviour during AP and C were compared using a Friedman test. To assess synchrony, spearman’s correlations were used to determine whether within-brood subjects’ and observers’ behavioural responses during the AP and C were correlated. Additionally, to determine overall brood behavioural synchronisation, for each brood we counted the number of observation points (max of 10) all members of the brood (n = 5) were performing the same behaviour during the treatment period for both AP and C. These were then compared using a Wilcoxon signed rank test.

Data were analysed using IBM SPSS Statistics 24. All data were checked for normality using a Kolmogorov-Smirnov test.

### Data availability

The data for this project will be openly available on the University of Bristol Research Data Repository.

## Results

### Eye temperature

There was a significant main effect of treatment (Wilks’s Lambda = 0.758, *F*_1,34_ = 10.864, *p* = 0.002, partial eta-squared = 0.242) and period (Wilks’s Lambda = 0.439, *F*_1,34_ = 43.402, *p* < 0.001, partial eta-squared = 0.561) and a significant interaction effect between treatment and period (Wilks’s Lambda = 0.307, *F*_1,34_ = 76.758, *p* < 0.001, partial eta-squared = 0.693). Specifically, both subjects and observers showed a decrease in eye temperature during to AP to subject, but not during the control test (Fig. 1).

There were no main effects of order (*F*_1,34_ = 0.204, *p* = 0.654, partial eta-squared = 0.006) or identity (*F*_1,34_ = 0.468, *p* = 0.499, partial eta-squared = 0.014) nor any interaction effects between treatment and order (Wilks’s Lambda = 0.985, *F*_1,34_ = 0.532, *p* = 0.471, partial eta-squared = 0.015), treatment and identity (Wilks’s Lambda = 1.000, *F*_1,34_ = 0.006, *p* = 0.937, partial eta-squared = 0.000), period and order (Wilks’s Lambda = 0.999, *F*_1,34_ = 0.028, *p* = 0.867, partial eta-squared = 0.001), period and identity (Wilks’s Lambda = 0.990, *F*_1,34_ = 0.354, *p* = 0.556, partial eta-squared = 0.010) and order and identity (*F*_1,34_ = 0.349, *p* = 0.559, partial eta-squared = 0.010).

**Figure 2:**
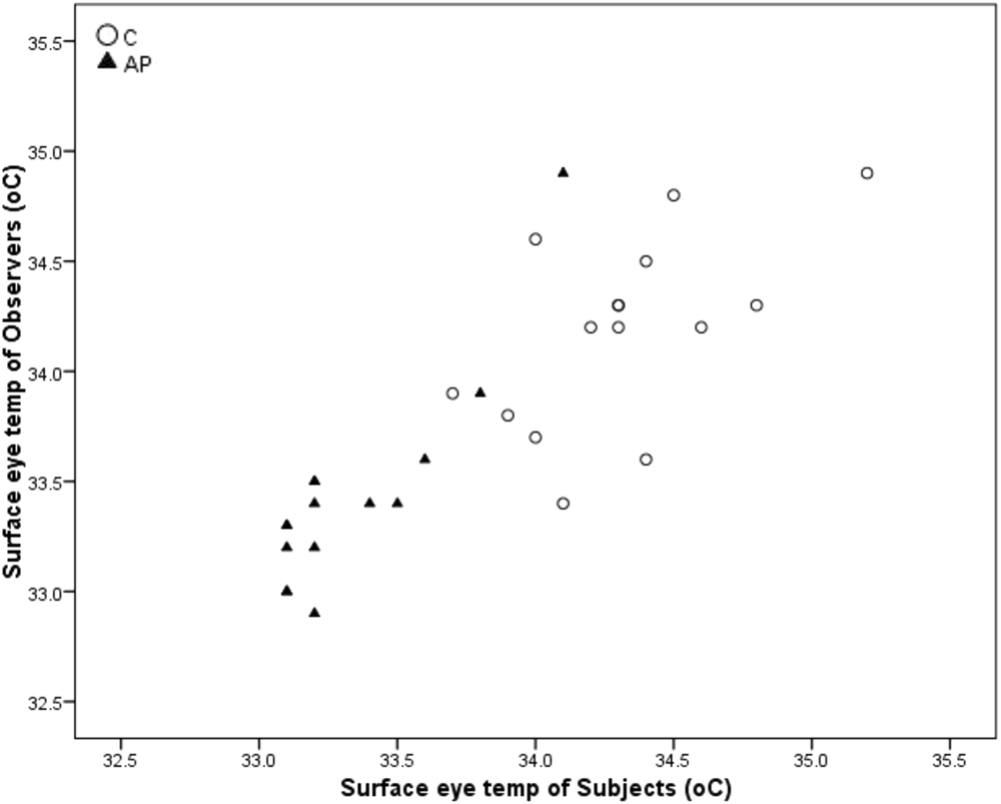
Correlations between Subjects’ and Observers’ Eye temperature during Control (C) and Air puff (AP).

A pearson correlation revealed a significant positive correlation between subjects’ and observers’ eye temperature responses during the AP (p < 0.001, r = 0.755, see Fig. 2) and C (p = 0.001, r = 0.688).

### Behaviour

Both subjects and observers decreased ground pecking and preening and increased freezing during the AP treatment period compared to the pre-treatment period. There were no such changes observed during the C (see Table 1 for statistics).

**Table 1:** Behavioural responses of subjects and observers during the tests (different letters after medians indicate significant differences, ns = not significant).

Behaviour	Identity	X^2^	df	Medians				highest posthoc p value
				C Pre-treat	C Treat	AP Pre-treat	AP Treat	
Ground peck	Subjects	31.984	3	30^a^	35^a^	26.67^a^	0^b^	<0.001
	Observers	37.652	3	30^a^	35^a^	40^a^	0^b^	<0.001
Stand	Subjects	3.435	3	35	35	37	40	ns
	Observers	8.765	3	35	35	40	50	ns
Sit	Subjects	5.483	3	15	11	13	7	ns
	Observers	4.473	3	15	15	10	5	ns
Preen	Subjects	30.529	3	17^a^	17^a^	10^a^	0^b^	0.001
	Observers	28.481	3	15^a^	15^a^	10^a^	0^b^	0.001
Freeze	Subjects	57	3	0^a^	0^a^	0^a^	40^b^	<0.001
	Observers	57	3	0	0	0	30^b^	<0.001
Walk	Subjects	4.5	3	5	5	0	0	ns
	Observers	2.589	3	5	5	0	0	ns

**Figure 3:**
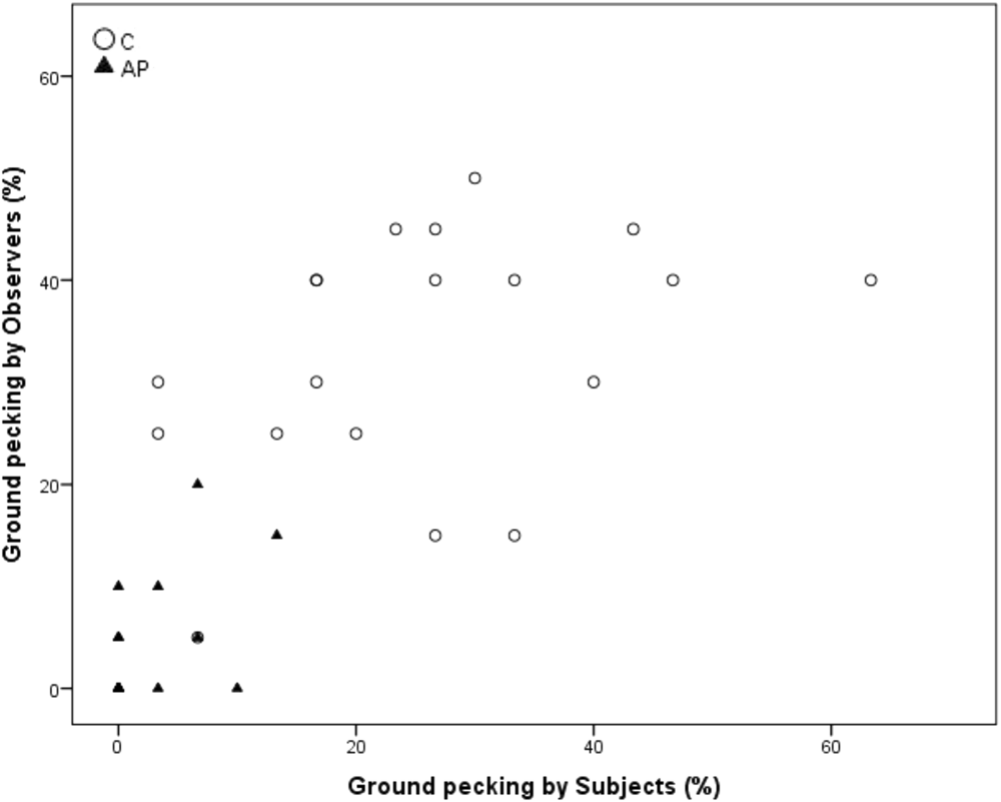
Correlations between Subjects’ and Observers’ Ground pecking during Control (C) and Air puff (AP).

**Figure 4:**
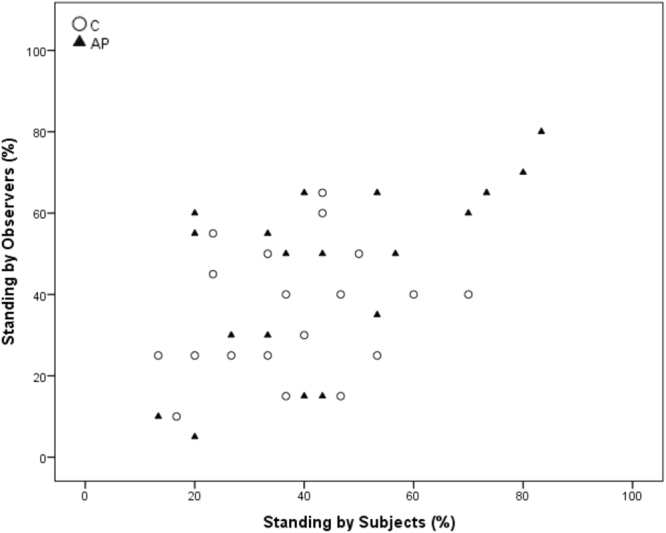
Correlations between Subjects’ and Observers’ Standing during Control (C) and Air puff (AP).

**Figure 5:**
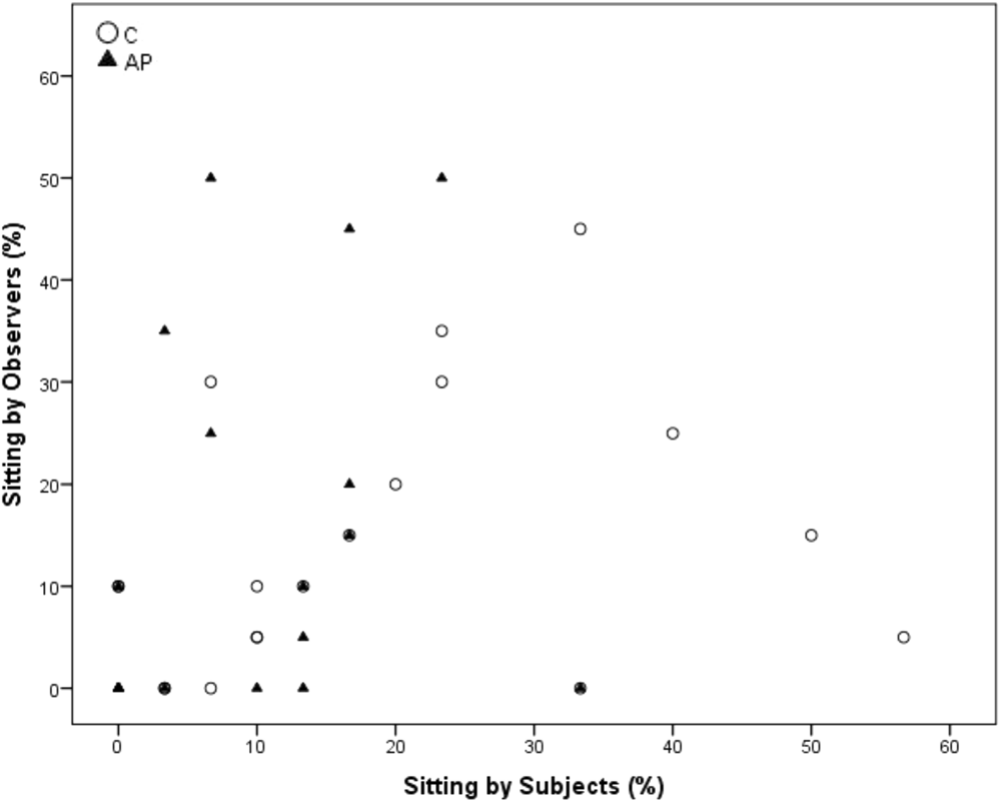
Correlations between Subjects’ and Observers’ Sitting during Control (C) and Air puff (AP).

**Figure 6:**
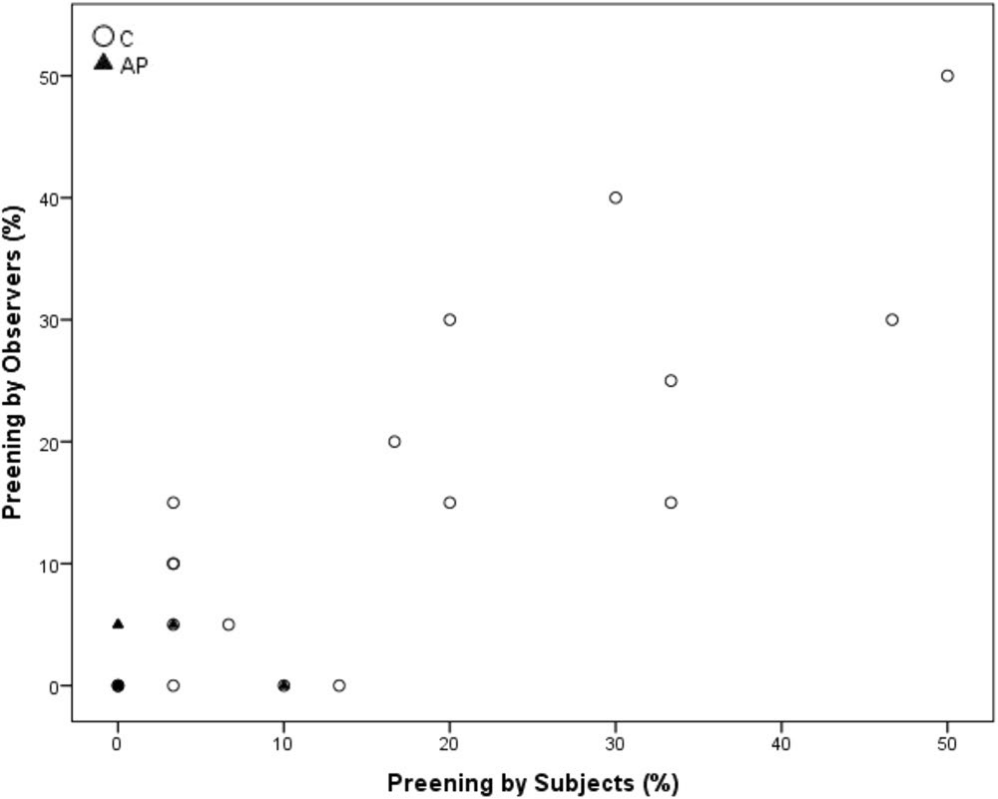
Correlations between Subjects’ and Observers’ Preening during Control (C) and Air puff (AP).

**Figure 7:**
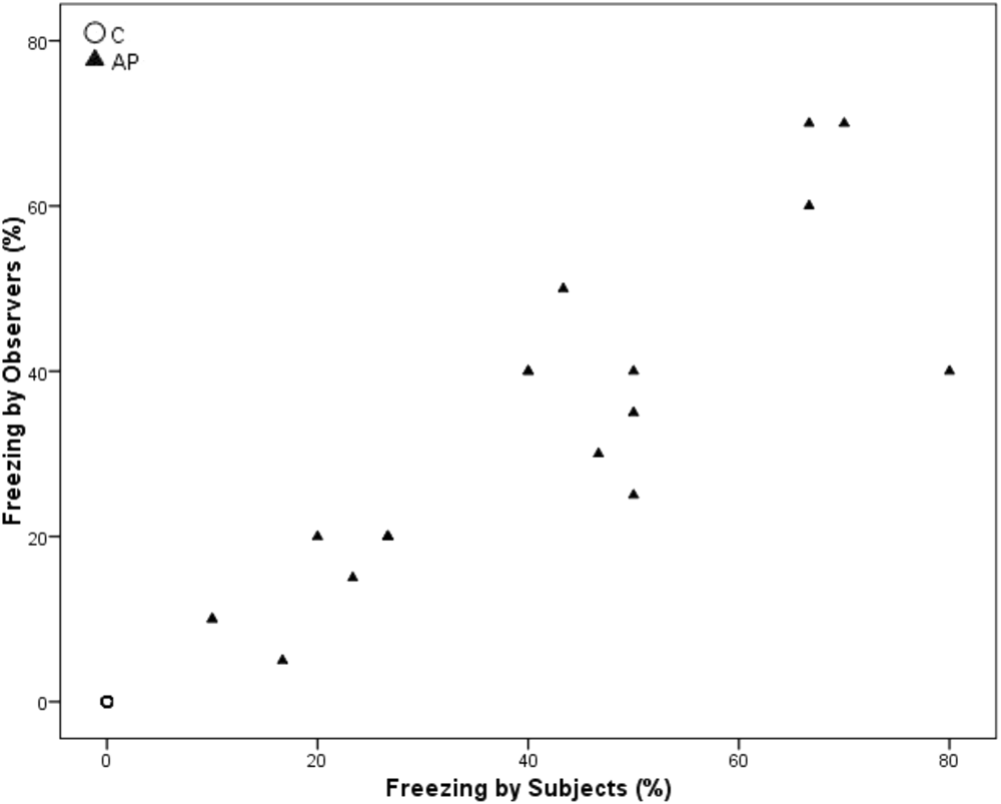
Correlations between Subjects’ and Observers’ Freezing during Control (C) and Air puff (AP).

**Figure 8:**
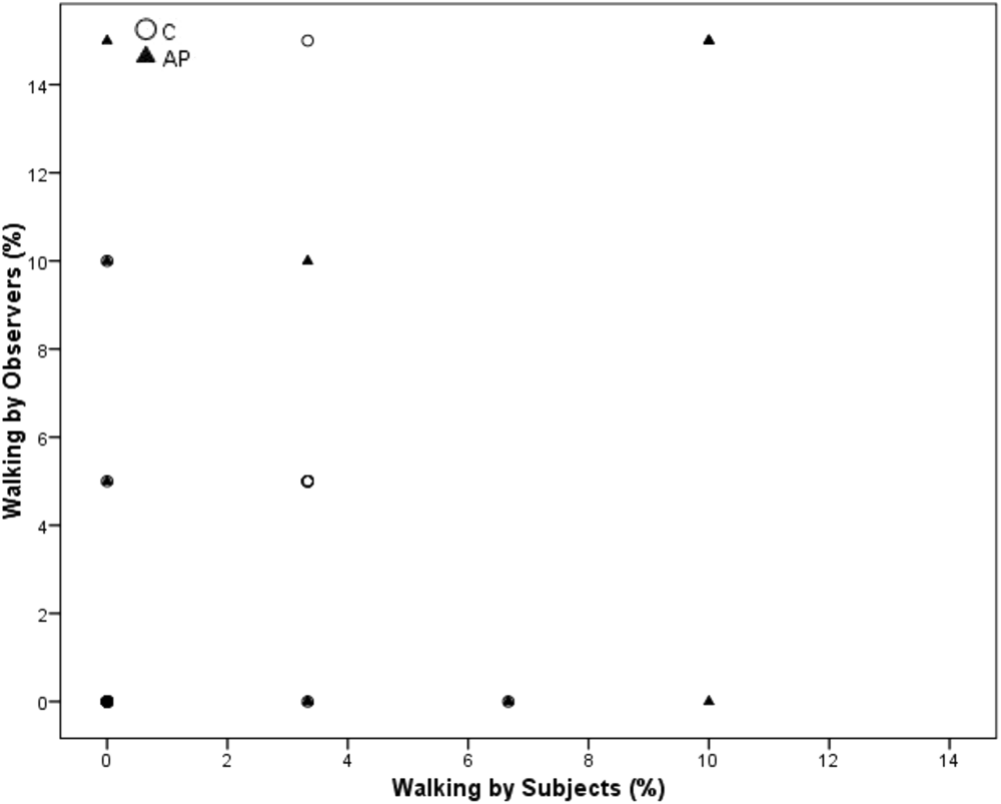
Correlations between Subjects’ and Observers’ Walking during Control (C) and Air puff (AP).

**Figure 9:**
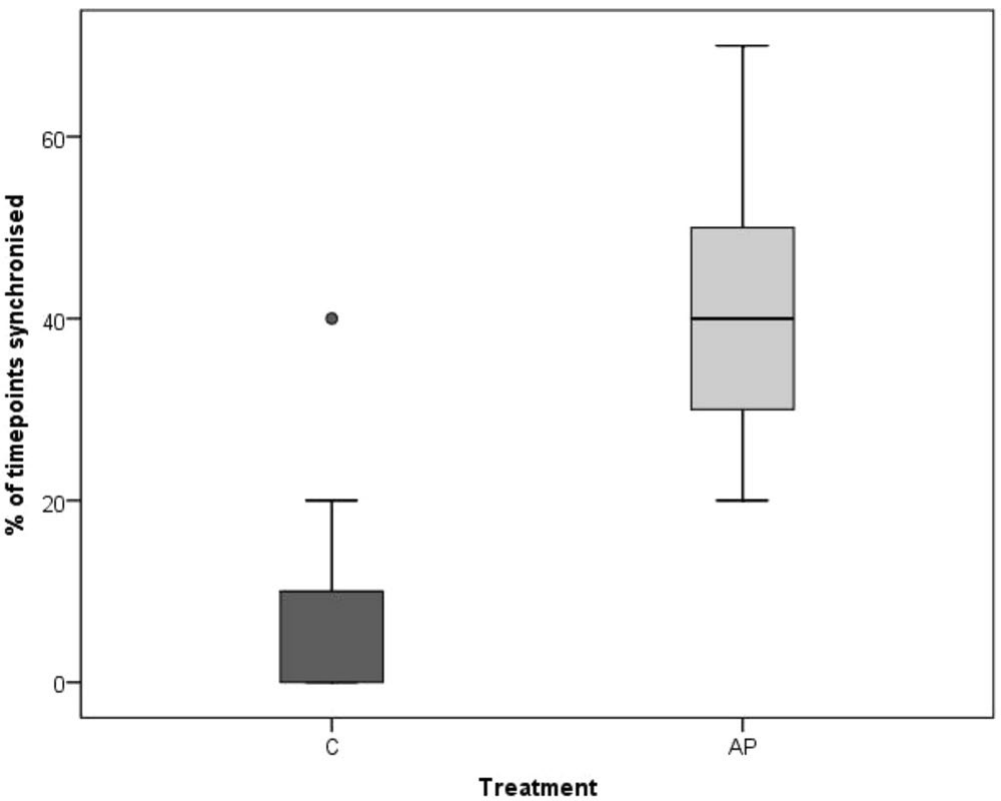
Percentage of timepoints all members of the brood were synchronised in their behaviour (‘°’ outliers >1.5 × IQR).

Spearman’s correlations revealed a significant positive correlation between within-brood subjects and observers in time spent ground pecking and standing during AP (Ground pecking p = 0.028, r = 0.502; standing p = 0.007, r = 0.597) but not C (Ground pecking p = 0.079, r = 0.413 standing; p = 0.318, r = 0.242). Freezing was significantly positively correlated during AP (p < 0.001, r = 0.872), but was entirely absent during the C condition. Preening was significantly correlated during C (p < 0.001, r = 0.796) but not during AP (p = 0.218, r = 0.297). Sitting and walking were not significantly correlated during C (Sitting: p = 0.104, r = 0.385; Walking p = 0.054, r = 0.450) or AP (Sitting: p = 0.056, r = 0.445; Walking p = 0.458, r = 0.181)) (see Figs 3–8 for scatterplots). Wilcoxon signed rank tests revealed that the whole brood spent a higher percentage of the timepoints showing the same behaviours during the AP than the C condition (z = −3.692, p < 0.001) (Fig. 9).

## Discussion

We have found that domestic chicks show socially-mediated arousal (SMA) and an increase in behavioural contagion when they witness the mild stress of their brood mates. Both subjects and observers showed decreased ground pecking and preening and increased freezing during the AP treatment, indicating an increase in arousal. Within the Subject-Observer brood pairings, standing, ground pecking and freezing were strongly positively correlated only during the AP condition. Preening was only positively correlated between Subject-Observer brood-pairings during the Control condition. Including all types of behaviour, the brood members were more behaviourally synchronised during the AP than the C condition. The correlated behavioural patterns between subjects and observers indicates that domestic chicks show behavioural synchrony of particular behaviours during both baseline and stressful situations, but that behaviour becomes more synchronised during the subject’s stressful situation.

Both subjects and observers also showed a reduction in eye temperature in response to AP. Additionally, eye temperature was strongly positively correlated between the 19 Subject-Observer brood-pairings during both the C and AP conditions; but more strongly so during AP. A reduction in surface temperature is indicative of arousal in birds and mammals; previously shown in adult hens during putative negative stressors such as handling^38^, witnessing chick stress^19^ and receiving air puffs^21^, as well as during anticipation of a positive food reward^39^. Domestic chicks also show this eye temperature reduction, although this is ameliorated by the addition of artificial maternal cues (maternal “cluck” call playback)^40^. Physiological synchrony of another feature of the HPA axis – cortisol – occurs between bonded individuals in humans (parents and their infants^30,31,41^ and pair bonded partners^42^) and songbirds (pair-bonded individuals^32^). More recently, this cortisol covariation was shown outside of such close social bonds, in cohabiting teleost fish^33^. Our results demonstrate that stress-induced hyperthermia, which can be measured both remotely and non-invasively, also shows this covariation.

Taken together, the changes in behaviour and eye temperature when observers witness the stressor applied to the subjects is indicative of SMA, with an associated increase in behavioural and physiological synchrony within the brood. Although we did not intend to study the valenced component of emotional contagion, indirect evidence from previous studies suggest that the responses observed are likely to be associated with a valenced emotional response; a prerequisite of empathy. Lower levels of preening have been observed by hens in behaviourally restrictive housing^43,44^. Hens also selectively avoid environments associated with high levels of standing alert and low bout durations of preening^45^, adding weight to the argument that the observer chicks showed a negatively valenced response to the stress of their broodmates.

The control condition, in which the air puff was sprayed from the same position but in the other direction induced no behavioural or eye temperature responses in the subjects and observers, indicating that the noise itself had not become a conditioned stimulus, nor did the extraneous features of the air puff such as the noise and smell have any measurable effect on the chicks during the AP condition. This replicates the findings of two of our previous studies where null responses were observed in similar control conditions^19,21^. During the control condition we found that chicks did show some synchronisation of behaviour and physiology. Namely, preening and eye temperature were both synchronised during the C condition. Preening and eye temperature may be closely linked; Buijs *et al*.^46^ found that eye temperature was strongly positively correlated to time spent preening, and suggested that the increased temperature might have been due to the physical activity of preening, a low head position or heat dissipation from the skin (e.g. underneath the wings). Since longer durations of preening occur in more positively valenced situations^45^ it is also a possibility that both preening and increased eye temperature may be indicative of a more positive affective state. The behavioural and physiological synchrony under baseline conditions may reflect contagion of this valence but further research is needed to determine the influence of both social and non-social processes on this synchrony.

The SMA found by chicks in this study contrasts with the findings of our previous study where adult hens showed no measured behavioural or physiological responses to an air puff applied to a familiar adult conspecific. This discrepancy could be due to several methodological differences between the two experiments. Firstly, the adult hens were tested in pairs, with one observer watching one subject. As a group-living species, chickens may be more likely to respond to more salient cues arising from several conspecifics, rather than just one. Secondly, the adult hens in the previous study were introduced to one another four weeks before the testing commenced, whilst the chicks in the current study were hatched and raised together for nine weeks. Familiarity was therefore higher in the current study and, akin to that of related members of a brood. Mice, observing a conspecific in pain increased their own pain-related behaviour only if the conspecific was a familiar cagemate^47^ or a close relative^18^ and familiarity is known to increase yawn contagion in animals^3^. Thirdly, in the current study, the chicks, as a whole brood, had received prior experience of the air puff, on two occasions five weeks previously, which may have enhanced their response to conspecifics receiving the same treatment. Studies into the effects of *individual* previous experience on responses of observers have yielded mixed results. Hens exposed individually (but in the presence of non-exposed conspecifics) to air puffs showed no greater response than naïve hens to chicks^19^ and hens^21^ exposed to the same stressor. In contrast, in pigs, previous individual experience of restraint enhanced the behavioural responses of observers to the restraint of a conspecific^8^. The speculated mechanism for this would be a recollected experience which is then applied to the conspecific’s situation. Further, if animals receive prior experience as part of a group, the behavioural response of conspecifics could become a conditioned stimulus predicting observer experience. In the current study, after only two prior exposures any such effect might be relatively weak but it cannot be discounted. Future studies should focus on systematically determining the extent of influence of both individual and group prior experience on SMA.

To conclude, this study demonstrates the co-occurrence of socially mediated arousal with physiological and behavioural contagion; all component features, (along with valence; which we did not intend to measure) of emotional contagion. To clarify the mechanisms involved, future work should examine the extent to which factors such as observer age, familiarity and prior exposure might underpin the stark contrast between the current results and the lack of effect previously observed in adult hens^21^.

## Electronic supplementary material

Supplementary information
