# Supplementary material for: Socially-mediated arousal and contagion within domestic chick broods
Source: Sci Rep. 2018 Jul 12;8:10509. doi: 10.1038/s41598-018-28923-8 (PMC6043517; doi:10.1038/s41598-018-28923-8)
Supplement: Supplementary file 1 — Supplementary information [file 41598_2018_28923_MOESM1_ESM.pdf]

Socially-mediated arousal and contagion within domestic chick broods

Joanne L. Edgar[1] and Christine J. Nicol[2]

[1]Bristol Veterinary School, University of Bristol, Langford House, Langford, BS40 5DU, UK
[2]Royal Veterinary College, Hawkshead Lane, North Mimms, Herts, AL9 7TA, UK

Corresponding author: j.edgar@bristol.ac.uk

Supplementary information

Animal and housing

19 broods of five female chicks (Lohmann classic) were each hatched under a broody hen (Australorp x Indian game). The chicks were unrelated Lohmann classic, obtained as eggs from a commercial hatchery and placed underneath the broody hens four days prior to hatching. Each hen and her brood of chicks were housed in a floor pen (1.5m x 1m) which was bedded with 5cm of wood shavings, and contained a feeder containing chick crumb and a chick drinker, allowing *ad libitum* food and water. The temperature in the room was 20°C and the lighting schedule was 16L: 8D. The day after hatching, chicks were individually marked using blue and red stock marker on their back and/or tail feathers.

Habituation to test box (Days 14-22)

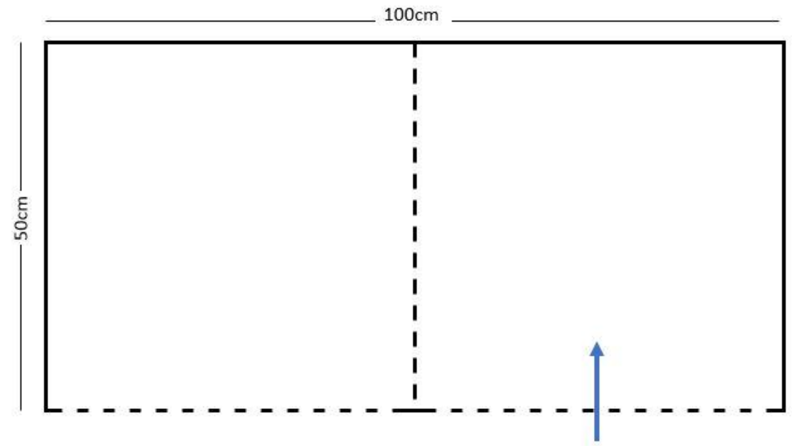

Fig Supp1. Test box apparatus showing the wire divider and position of air puff release during AP

Table Supp1. Habituation of chicks to test box

Day of age	Habituation session	Time in box (mins)
14	1	5
	2	10
15	3	5
	4	15
17	5	20
21	6	20
22	7	25

From days 20 to 22, chicks were exposed to the sight of a thermal video camera positioned on a tripod 1m from the test box.

Days 23-65

Immediately following habituation, the chicks experienced a further four 25-minute periods together as a brood in the observer box over 2 days, including two 10-minute exposures to air puffs (for 1s every 30s). After this, the chicks were handled weekly to obtain information on weight and physical health. On day 56, to coincide with the natural cessation of maternal care, the broody hens were removed from the pens and were rehomed.

Table Supp2. Behavioural ethogram

Behaviour	Description
Stand	Chick in upright posture with legs extended, without performing any other described behaviour
Sit	Chick with legs bent underneath body and breast against the floor, without performing any other described behaviour
Preen	Chick using beak to manipulate their own feathers

Ground peck/scratch	Chick using beak to manipulate the ground and/or using digging motion with legs
Walk	Chick moving around the test box in upright posture, without performing any other described behaviour
Freeze	Standing with eyes open and no body or head movement for at least five seconds
